# Revealing the Functional Microbiota of Caproic Acid-Producing and Lactic Acid-Utilizing Bacteria in the Pit Muds for Chinese *Nong-Xiang Baijiu* Fermentation

**DOI:** 10.3390/foods15030416

**Published:** 2026-01-23

**Authors:** Qingwei Feng, Xiaohan Li, Lijuan Gong, Yanxia Wei, Zhongxue Bai, Jian Zhou, Yi Ma, Guiqiang He

**Affiliations:** 1 Engineering Research Center of Biomass Materials, Ministry of Education, College of Life Sciences and Agri-Forestry, Southwest University of Science and Technology, Mianyang 621010, China; 2 Liquor Making Biotechnology and Application Key Laboratory of Sichuan Province, Sichuan University of Science and Engineering, Yibin 644000, China; 3 Liquor Making Biotechnology and Intelligent Manufacturing of Key Laboratory of China National Light Industry, Yibin 644000, China

**Keywords:** Chinese *baijiu*, pit mud, caproic acid, lactic acid, functional microbiota

## Abstract

Low ethyl caproate and high ethyl lactate contents pose a significant challenge in producing Chinese *nong-xiang baijiu*. The formation of these esters depends on the metabolism of their precursors—caproic acid and lactic acid—within the pit mud (PM) microbiome. However, the specific taxa driving the metabolic flux from lactate accumulation to caproate synthesis remain unclear. This study aimed to identify potential functional microbes capable of caproate biosynthesis and lactate utilization by systematically analyzing PM samples from the upper, middle, and lower layers of three different pit ages (0, 20, and 50 years). Results showed that 50-year-old PM exhibited significantly higher caproic acid and ammonium nitrogen levels, but lower lactic acid content, compared to the 0- and 20-year-old counterparts. Notably, *Petrimonas*, *Caproiciproducens*, and *Sedimentibacter* were significantly enriched in the 50-year-old PM. Their relative abundances correlated positively with caproic acid and negatively with lactic acid. Furthermore, PICRUSt2 analysis indicated higher abundances of genes associated with caproate synthesis and lactate utilization in the 50-year-old microenvironment. We propose that *Petrimonas*, *Caproiciproducens*, and *Sedimentibacter* are potential functional candidates for lactate degradation and caproate generation. These findings provide a scientific basis for modulating the microbiome for “increasing ethyl caproate and reducing ethyl lactate”, thereby enhancing *baijiu* quality.

## 1. Introduction

Chinese *baijiu* is a time-honored traditional fermented food, embodying millennia of culinary wisdom through its unique fermentation techniques. As a quintessential representative of Chinese *baijiu*, *nong-xiang baijiu* stands out globally, renowned for its distinctive sensory characteristics, which are characterized by a rich, persistent aroma, velvety texture, and harmonious sweetness [1,2]. This unparalleled flavor profile traces back to its thousands of years of brewing heritage, which prominently employs the “solid-state anaerobic fermentation in mud pits” [3,4]. Unlike other *baijiu* styles, this unique process relies on the metabolic activities of functional microbiota inhabited in pit mud, and these microorganisms can catalyze and synthesize over 1500 identified flavor compounds, with esters and acids constituting the major aroma contributors [1].

Pit mud (PM), as the core microbial ecosystem in *nong-xiang baijiu* traditional fermentation processes, plays a pivotal dual role that underscores its irreplaceable significance. Functionally, it provides an anaerobic niche essential for fermentation, while also serving as a reservoir for key microbiota. For example, PM was the sustained-release source of anaerobes, which accounted for over 14% of prokaryotic communities in fermented grain during the *nong-xiang baijiu* fermentation [5]. Moreover, metagenomic studies have revealed that dominant genera, such as *Clostridial* cluster IV, *Caloramator*, *Clostridium*, *Sedimentibacter*, *Bacteroides* and *Porphyromonas*, are responsible for producing caproic acid [6]. Specifically, metabolic interactions of PM microbes directly dictate the olfactory and gustatory profiles of *nong-xiang baijiu*, making PM a critical determinant of product quality [7].

In recent years, significant progress has been made in the study of PM. A significant body of research has been conducted on PM samples from different vintages, geographic locations, and enterprises. These studies have revealed substantial variations in pit microbial community structure [8]. For instance, the prevalence of functional flora, typified by caproic acid-producing bacteria, is observed to be elevated in century-old pits. Conversely, new pits are characterized by a preponderance of diverse bacterial species, including *Lactobacillus* [9]. Meanwhile, simulated fermentation experiments revealed that the content of ethyl caproate, in particular, was significantly higher in fermented grain samples supplemented with PM. Bacteria dominated by *Caproiciproducens*, *Caloramator*, *Sedimentibacter*, and *Caldicoprobacter* migrated from the pit mud to the fermented grains [10]; however, the specific relationship between pit mud microorganisms and organic acids was not elaborated in detail. Although multi-omics technologies have been employed in previous studies to preliminarily elucidate the metabolic mechanisms of caproic acid-producing bacteria [3,11], maintaining microecological balance remains a major challenge in actual production. In particular, improper pit management or fluctuations in fermentation conditions often result in excessive lactic acid accumulation and subsequent acidification of the PM. This environmental stress strongly inhibits the metabolic activity of caproic acid-producing bacteria [11], ultimately compromising the flavor quality of *baijiu*, which is characterized by the defect of “lower caproic acid and higher lactic acid content”. Currently, the patterns of community interaction between lactic acid-degrading bacteria and caproic acid-producing bacteria, their coordinated response mechanisms to environmental stress, and the associations between functional microbial communities in PM and organic acids remain incompletely understood under such imbalanced conditions. This has become a critical knowledge gap that hinders the achievement of quality regulation targeting “increasing ethyl caproate and reducing ethyl lactate”.

Given the aforementioned challenges, this study aimed to systematically investigate the community structural characteristics and ecological associations of lactic acid-degrading bacteria and caproic acid-producing bacteria in PM of *Nong-xiang baijiu*. Specifically, this study analyzed the distribution patterns of these two core functional microbial groups across different PM samples in depth and elucidated their symbiotic or competitive mechanisms within the complex fermentation system by integrating correlation analysis with environmental factors. The findings of this study will provide a novel perspective for uncovering the maintenance mechanisms of PM microecological functions, and offer a scientific basis for addressing the bottleneck problem of “lower caproic acid and higher lactic acid content” in practical production and improving the theoretical system of microbial brewing for *Nong-xiang baijiu*.

## 2. Materials and Methods

### 2.1. Sample Collection

**Figure 1:**
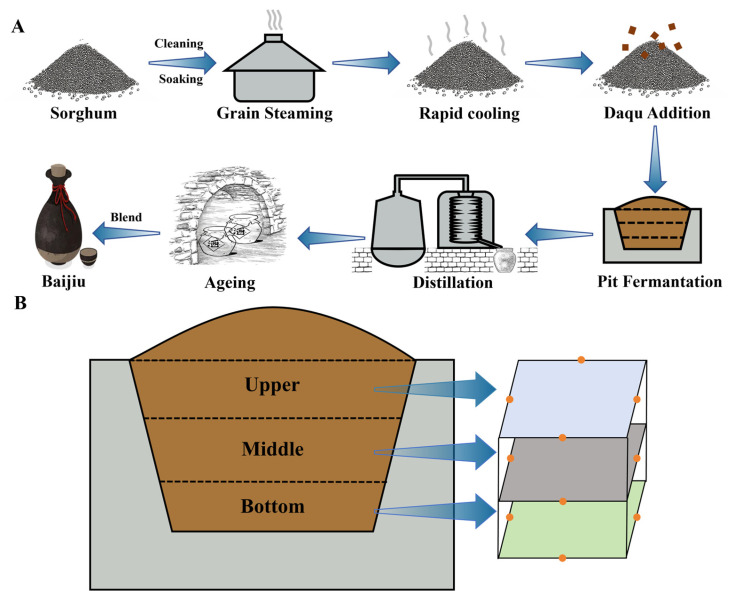
Explanation of PM sampling sites. (**A**) Schematic diagram of the *nong-xiang baijiu* brewing process. (**B**) Schematic illustration of PM sample collection (including sampling layers and points).

PM samples of varying vintages were obtained from a *nong-xiang baijiu* enterprise in Sichuan Province, China. PM samples were taken from pits that had been used continuously for 0, 20, and 50 years, with the upper, middle, and bottom layers taken from each pit. To ensure the representativeness and spatial coverage of the samples, a stratified multi-point mixed sampling strategy was adopted: in the upper, middle and bottom layers of each pit, four sampling sites were set up in the center of the pit wall on all sides, and PM samples at a depth of 0–5 cm were collected at each site (200 g/site). Three parallel replicates were collected at each sampling point, resulting in a total of 27 samples, and a schematic diagram of the sampling sites can be found in Figure 1. The samples from these positions were then subjected to homogenization and numbered sequentially. Thereafter, they were transferred to a sterile sample bag and stored at −80 °C. The uppermost layer of PM was designated 0-U, 20-U, and 50-U, while the intermediate layer was numbered 0-M, 20-M, and 50-M. Correspondingly, the lowermost layer of PM received the designations 0-B, 20-B, and 50-B.

### 2.2. Determination of the Physical and Chemical Properties of PM

The moisture content of PM was measured using the dry/wet gravimetric method, and the samples were dried at 105 °C for 3 h. To determine pH, the PM samples and water were mixed intermittently for 30 min in a ratio of 1:10, and then measured using a pH meter (PHS-3E, SPSIC, Shanghai, China). Determination of NH_4_^+^-N and available phosphorus in PM was performed according to the method of Xia, et al. [4]; ammoniacal nitrogen (NH_4_^+^-N) in the PM samples was leached out with a 10% NaCl solution, was reacted with Nye’s reagent, and then the absorbance was measured at 425 nm using an ultraviolet spectrophotometer (UV-5500PC, Metash, Shanghai, China). Effective phosphorus in the PM samples was extracted using NH_4_F-HCl solution and the absorbance was measured at 680 nm after reaction with acidic ammonium molybdate solution. The content of four organic acids (lactic acid, acetic acid, butyric acid and caproic acid) in the PM was determined by high-performance liquid chromatography (HPLC), according to the method of Chen, et al. [12], using a 300 mm × 6.5 mm Alltech OA-1000 column (Agilent, Santa Clara, CA, USA) with a mobile phase of 9.00 mM H_2_SO_4_ at a flow rate of 0.60 mL/min, and a sample volume of 15 μL. The temperature of the oven was 65 °C, and the detection wavelength was 210 nm.

### 2.3. DNA Extraction, PCR Amplification, and Sequence Analysis

Total genomic DNA samples were extracted using the OMEGA Soil DNA Kit (M5636-02) (Omega Bio-Tek, Norcross, GA, USA), following the manufacturer’s instructions, and stored at −20 °C prior to further analysis. The quantity and quality of extracted DNAs were measured using a NanoDrop NC2000 spectrophotometer (Thermo Fisher Scientific, Waltham, MA, USA) and agarose gel electrophoresis, respectively. The V3-V4 region of bacterial 16S rRNA in PM samples was amplified using forward primer 338F (5′-ACTCCTACGGGGAGGCAGCA-3′) and reverse primer 806R (5′-GGACTACHVGGGTWTCTAAT-3′). Sample-specific 7-bp barcodes were incorporated into the primers for multiplex sequencing. The PCR parameters were set according to the previously established protocol [13]. PCR amplicons were purified with Vazyme VAHTSTM DNA Clean Beads (Vazyme, Nanjing, China) and quantified using the Quant-iT PicoGreen dsDNA Assay Kit (Invitrogen, Carlsbad, CA, USA). After the individual quantification step, amplicons were pooled in equal amounts, and pair-end 2 × 250 bp sequencing was performed using the Illumina NovaSeq platform with NovaSeq 6000 SP Reagent Kit (500 cycles) at Shanghai Personal Biotechnology Co., Ltd. (Shanghai, China).

The microbial bioinformatics analysis was conducted using QIIME2 2019.4 software [14]. The original sequence data was decoded and processed using the Demux plugin, followed by primer sequence removal via the cutadapt plugin. To effectively eliminate PCR artifacts, low-quality sequences, and chimeras, the DADA2 plugin was subsequently applied for sequence quality filtering, denoising, paired-end sequence assembly, and chimera detection and removal. Only non-singleton amplicon sequence variants were retained to further exclude low-abundance artifacts. Following the removal and screening of the obtained sequences, they were then merged based on 100% sequence similarity. This process results in the generation of sequences that possess specific features, which are known as Amplification Sequence Variants (ASVs) and Abundance Data Tables. For taxonomic annotation, ASV feature sequences were aligned against reference sequences in the SILVA Release 132 database to obtain taxonomic information for each ASV. Functional prediction analysis relied on the MetaCyc and KEGG databases.

### 2.4. Data Analysis

Single-factor analysis of variance (ANOVA) was utilized to evaluate the significant differences in each component of PM (*p* < 0.05). All samples in this study were prepared with three independent biological replicates (i.e., three parallel samples were collected from each sampling site and subjected to separate experimental analyses) to ensure data reliability and the validity of statistical tests. A sparse curve was drawn to evaluate the validity of the sequencing data of PM samples. Alpha diversity indices such as Chao1, ACE, Shannon, and Simpson were calculated using the ASVs table in QIIME and visualized by box plots. Between-group differences in abundance classification units were detected using LEfSe linear discriminant analysis effect size [15]. Differences in microbial community structure were demonstrated by non-metric multidimensional scaling (NMDS) and hierarchical cluster analysis [16]. The present study investigates the correlation between the microbial community associated with “caproic acid-producing and lactic acid-utilizing” bacteria and four organic acids. To this end, Redundancy Analysis (RDA) is performed using Canoco 5.0 software. As indicated by the KEGG PATHWAY database, the potential functions related to the organic acid metabolism of microbial communities in PM samples were predicted using PICRUSt2 v2.1.0-b [17].

## 3. Results and Discussion

### 3.1. Analysis of Physical and Chemical Properties of PM

**Figure 2:**
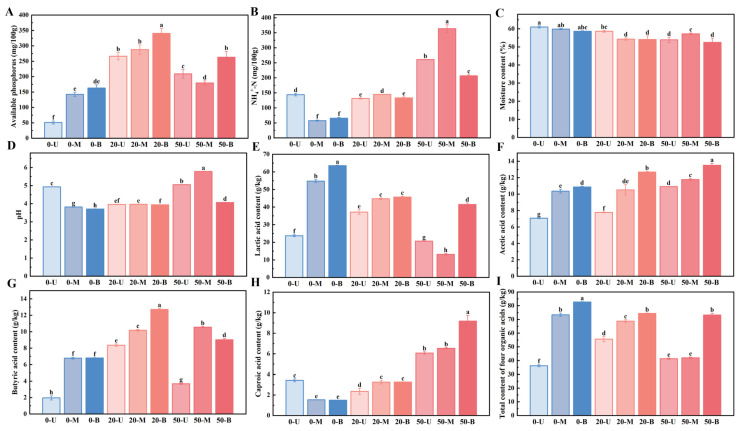
Physicochemical properties of PM samples with different characteristics. (**A**) Available phosphorus content. (**B**) NH_4_^+^-N content. (**C**) Moisture content. (**D**) pH value. (**E**) Lactic acid content. (**F**) Acetic acid content. (**G**) Butyric acid content. (**H**) Caproic acid content. (**I**) Total content of four organic acids (lactic acid, acetic acid, butyric acid, and caproic acid). Different letters indicate differences in data between groups (*p* < 0.05). Error lines indicate the standard deviation of three independently repeated experiments.

In the *nong-xiang baijiu* brewing system, PM plays a decisive role in the generation and proportion regulation of micro-flavor components as the core microecological carrier. Its physical and chemical properties are dynamically coupled with the microbial community structure and metabolic activity, which directly determines the accumulation efficiency of the main flavor components, such as ethyl caproic acid, and the equilibrium of the coordinating components, such as ethyl lactate, by influencing the abundance and metabolic pathway of the functional bacterial flora. We, therefore, measured the physicochemical properties of different PM samples, including pH, moisture content, effective phosphorus content, ammoniacal nitrogen content, and the content of the following organic acids: lactic acid, acetic acid, butyric acid, and caproic acid (Figure 2).

As the age of the pit grows, the physicochemical properties of the PM show significant differences. The 0-year-old PM has the highest moisture content, and at the same time, the moisture gradually decreases from the upper layer to the lower layer, reaching 60.98 ± 0.57, 59.81 ± 0.31% and 58.59 ± 0.32%, respectively; the 50-year-old PM has the lowest average moisture, and the difference in moisture content of the layers is not significant. pH, as a factor that directly affects microbial growth and metabolism, is the highest in the 50 years of PM; during this time, the middle layer of PM reaches the highest level 5.78 ± 0.01. In the brewing of *nong-xiang baijiu*, effective phosphorus and ammonium nitrogen in the PM have extremely critical effects on microorganisms. The 20-year-old PM had the highest content of effective phosphorus, which is an important substance for synthesizing proteins and participating in the synthesis of caproic acid [18], which indicates that the microbial community in 20-year-old PM has a higher protein synthesis capacity. Similar to the results of others [4], the NH_4_^+^-N content gradually increased with the increase in pit age, especially in the middle layer of 50-year-old PM, which reached 363.67 ± 12.98 mg/100 g. In general, the above four physicochemical indicators were most special in the 50-year-old PM, and among them, the middle layer of 50-year-old PM differed significantly from that of the upper and the lower PM. The results of organic acid content analysis showed that the lactic acid content decreased significantly with the increase in pit age, as shown in Table S1 and Figure 2, which may be due to the enrichment of lactic acid-degrading bacteria in the fermentation system [19]. In addition, the lactic acid content in the middle part of the 50-year-old PM is the lowest, at 13.14 ± 0.44 g/kg. In contrast, the bottom of the pit contained a higher lactic acid content, which was common in the fermentation process. The main reason for this was that the bottom PM was affected by the high concentration of yellow water, which led to the high lactic acid content [20]. Butyric acid content first increased and then decreased with pit age, and was highest in the bottom layer of 20-year-old PM. In addition, acetic acid and caproic acid contents were enriched during the growth of pit age. From the total change trend of the four acids, the total content of the four organic acids gradually decreased. This is mainly because lactic acid, which had the highest initial content among the four acids, showed a marked downward trend with increasing pit age. Similar to the research results of Zhang et al. [21], lactic acid content was lower in aged PM. Although the contents of the remaining three organic acids showed an increasing trend, their total increment was smaller than the decrease in lactic acid.

### 3.2. Analysis of Bacterial Diversity in PM

**Figure 3:**
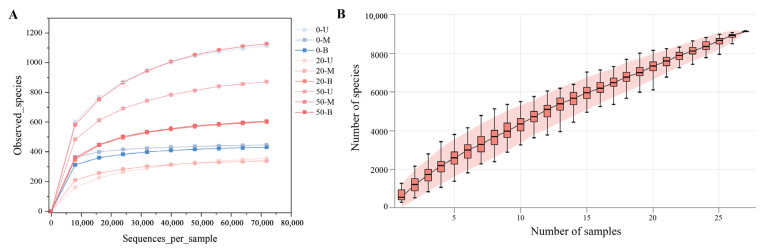
Rarefaction and species accumulation curves of PM microbial communities. (**A**) Rarefaction curves showing the number of observed species in PM samples. (**B**) Species accumulation curve showing the trend of species number increase with the expansion of PM sample size, with the shaded area representing the 95% confidence interval.

Following the removal of primers, low-quality sequences, denoising, and chimerism, the samples were clustered with 100% similarity. Thereafter, DADA2 quality control was utilized to obtain the ASV sequences of each sample. As demonstrated in Table S1, the numbers of high-quality sequences obtained from 0-year-old PM, 20-year-old PM, and 50-year-old PM are 88,268–89,506, 119,007–130,054, and 91,137–100,869, respectively. The ratio of high-quality sequences to effective sequences in the vast majority of PM samples exceeds 90%. Furthermore, the bacterial diversity detected was used to construct a sparse curve (Figure 3A). It was established that as the sequencing depth attained 47,897, the sparse curve underwent gradual stabilization, signifying that at this juncture, with the augmentation of the sequencing depth, the detection of novel species became infrequent. It is evident that, on account of the sequencing depth of 71,845, which is considerably greater than that of 47,897, the coverage rate surpassed 99%. Consequently, the sequencing data presented in this study can be considered representative of the majority of bacterial communities present in the PM sample. Concurrently, the species accumulation curve (Figure 3B) was plotted, which demonstrated that as the sample size increased, the number of observed species also increased. The curve demonstrates a precipitous increase at the inception, signifying that, with a reduced number of initial samples, the identification of novel species for each subsequent sample is enhanced. As the sample size continues to increase, the gradient of the curve gradually decreases, indicating that with each additional sample, the number of newly discovered species decreases in proportion. Despite the upward trend observed in the final samples, the gradient became negligible, suggesting that, at the current sample size, the majority of species were identified, though a small number of undiscovered species may still be present. Based on the three pit samples available, the conclusions drawn are limited to the community characteristics and stratification patterns within the same-aged PMs, and it is not possible to infer community differences between the same-aged PMs for the time being due to the limitation of the number of pits.

**Figure 4:**
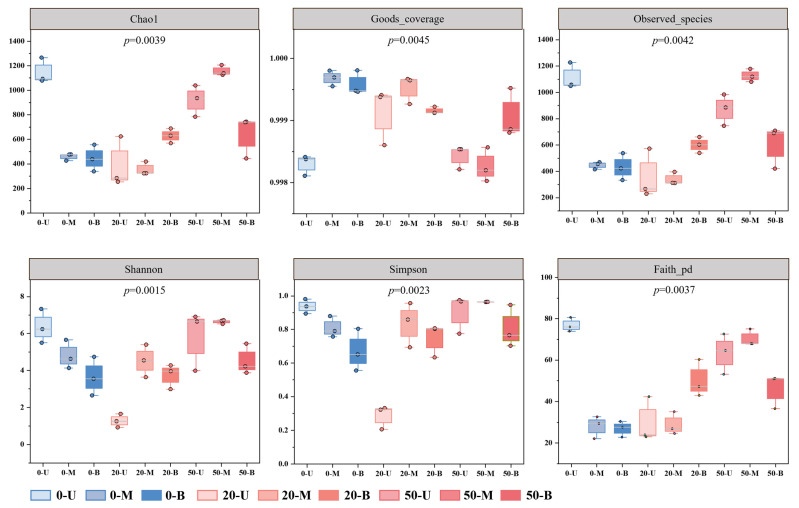
Alpha diversity indices of microbial communities in different PM samples.

Alpha analysis constitutes a pivotal component within the broader framework of microbial diversity analysis. In order to comprehensively evaluate the alpha diversity of PM microbial communities, the following indices were calculated: Chao1, Goods_coverage, Observed_species, Shannon, Simpson, and Faith_pd. The purpose of this was to characterize the diversity, richness, and coverage of bacterial communities in PM (Figure 4, Table S2). The Chao1 index was positively correlated with species richness, which increased with the age of the PM, and it is particularly noteworthy that the species abundance in the middle layer of the 50-year-old PM showed higher levels. The reason for this phenomenon may be due to the gradual enrichment and diversification of the microbial community in the PM as the age of the pit increases. It is an established fact that during the long-term fermentation process, different types of microorganisms may gradually adapt and reproduce under specific environmental conditions, thus leading to an increase in species abundance. For 50-year-old PM, the prolonged fermentation duration has likely stabilized the microbial community. During this period, various microorganisms have undergone more extensive growth and development, leading to higher species abundance. Furthermore, it is important to consider that changes in temperature, humidity, and oxygen concentration, amongst other factors, can also affect the diversity and abundance of microbial communities within the pit environment. The emergence of these differences also indicates that the microbial community within PM is constantly evolving and dynamic.

### 3.3. Analysis of Bacterial Species Differences and Marker Species

In order to further explore the composition pattern of PM microbiota, the feature table was subjected to statistical analysis after removing singletons, and the microbial composition in the samples was analyzed at the phylum and genus levels. The ten most abundant microbial species were illustrated using stacked bar charts.

**Figure 5:**
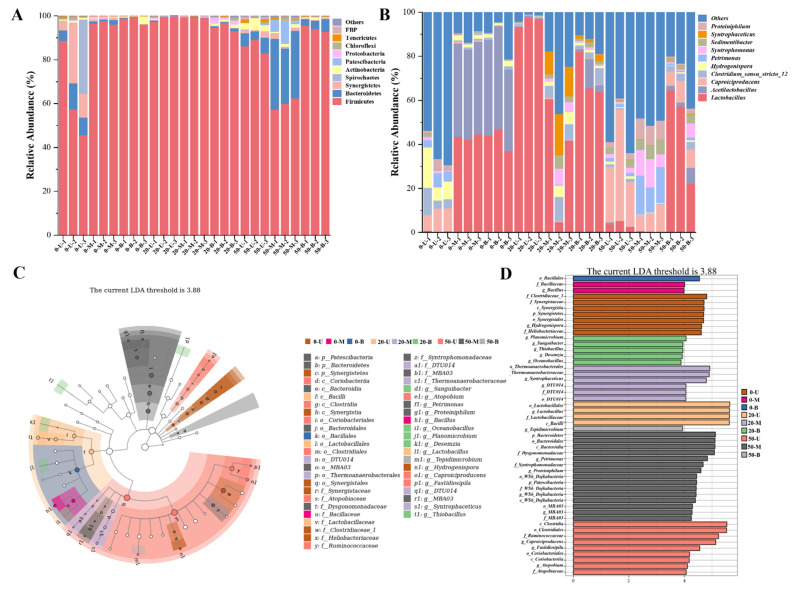
Species composition of bacterial communities in different PM samples. (**A**) Species composition of PM microbial community at the phylum level. (**B**) Species composition of PM microbial community at the genus level. (**C**) The differentiability of bacterial communities is represented by the effect size of linear discriminant analysis (LEfSe) (LDA > 3.88, *p* < 0.05). (**D**) The bar chart displays the characteristic bacterial communities in each sample based on LEfSe analysis (LDA > 3.88, *p* < 0.05).

As demonstrated in Figure 5A, at the phylum level, the PM samples were predominantly composed of Firmicutes and Bacteroidetes. Significant differences were observed in the composition and structure of microbial communities across different layers of PM. For instance, in the upper layer of 50-year-old PM, Firmicutes exhibited the highest abundance (82.99–89.23%), followed by Bacteroidetes (3.77–7.20%) and Actinobacteria (2.82–5.43%); in the middle layer of 50-year-old PM, Firmicutes (57.34–62.41%), Bacteroidetes (25.17–32.31%), and Patescibacteria (1.08–10.29%) emerged as the dominant phyla; and in the bottom layer of 50-year-old PM, the main constituent phyla were Firmicutes (92.83–95.64%) and Bacteroidetes (3.10–5.81%). Concurrently, an increase in the age of the pit was observed to result in the accumulation of Bacteroidetes and Patescibacteria, alongside a decline in Firmicutes. This phenomenon may be attributable to the influence of the fermentation environment and biological evolution, which can result in the degradation of PM, a common occurrence during the maturation and succession of PM [22,23]. It is evident that the predominant bacterial communities in PM and yellow water are reported to be Firmicutes, Bacteroidetes, Actinobacteria, and Proteobacteria [24,25].

At the genus level (Figure 5B), the microbial populations with higher richness in PM are *Lactobacillus*, *Acetilactobacillus*, *Caproiciproducens*, *Clostridium*, and *Syntrophomonas*. As the age of the pit increases, there is a gradual increase in species richness, which is consistent with the results of the alpha diversity analysis. The alterations in species abundance observed in the 0-year-old PM indicate the presence of a greater number of microbial species on the upper layer of the PM. This finding suggests that the dominant species had not yet formed in the new PM, indicating that the upper PM is more unstable at this time. Lactic acid and ethyl lactate have been identified as the key flavor compounds in the fermentation process of *nong-xiang baijiu*. However, it is important to note that excessive content can potentially disrupt the flavor equilibrium of *nong-xiang baijiu*, thereby compromising its overall quality. *Lactobacillus*, a producer of significant quantities of lactic acid in PM, also plays an important role in carbohydrate metabolism, protease production, and flavor improvement [26,27]. This strain is prevalent during the initial phase of fermentation. However, as the age of the pit increases, there is a concomitant decrease in the abundance of *Lactobacillus*, both on the pit wall and on the pit floor, with a concomitant increase in the abundance of *Caproiciproducens*, *Syntrophomonas*, and *Petrimonas*. It is imperative to exercise meticulous control over the content of *Lactobacillus* during the fermentation process in order to regulate the content of lactic acid in *nong-xiang baijiu* [28]. Over time, we found that *Sedimentibacter* accumulates gradually, and *Sedimentibacter* has the effect of degrading lactic acid [29], and its accumulation in PM provides an effective way to regulate lactic acid during the production of *nong-xiang baijiu*. *Caproiciproducens* as well as *Petrimonas* are identified producers of caproic acid and butyric acid, the content of which is a key indicator for evaluating the quality of *nong-xiang baijiu* [30]. Furthermore, both genera have the highest content of PM in 50 years, with the abundance of *Caproiciproducens* reaching 6.97–50.70% and that of *Petrimonas* reaching 0.65–17.76%. This finding suggests a consistent trend of changes with pit age and the abundance of *Caproiciproducens* and *Petrimonas* [22,31]. Furthermore, *Petrimonas* is a microorganism of Bacteroidetes that can utilize glucose metabolism to produce H_2_, CO_2_, acetic acid, or propionic acid and can be fermented to produce short-chain fatty acids such as butyric acid and caproic acid [32,33]. Consequently, *Petrimonas* has made notable contributions to the promotion of PM maturation and the provision of flavor compounds. Furthermore, an analysis of the samples revealed a notable abundance of *Clostridiumsenss_tricto_12*, a microbe capable of producing caproic acid. This microbe was found to be most prevalent in the PM of 20 years, particularly in the PM of the middle layer of the pit in 20 years, with a content ranging from 5.49% to 10.59%. Previous studies have confirmed that the abundance of functional microorganisms is higher in aged PM [21,34], but the coordinated variation patterns of microorganisms associated with caproic acid production and lactic acid degradation during PM aging have not been clearly elucidated. By comparing PM samples of 0, 20, and 50 years of age, this study found that with increasing PM age, lactic acid content exhibited a decreasing trend, while caproic acid content and the abundances of related functional microorganisms (*Petrimonas*, *Caproiciproducens*, and *Sedimentibacter*) showed an increasing trend. The revelation of this dynamic evolutionary pattern clarifies the directional shaping effect of PM age on functional microbial communities and provides a critical theoretical basis for the acclimatization of artificial PM and the functional reconstruction of aged PM.

The LEfSe plot (Figure 5C,D) is utilized for the purpose of visualizing microbial communities that exhibit significant disparities amongst disparate groups. It is imperative to note that the microbial groups exhibiting LDA values greater than 3.88 are incorporated into the figure, with the objective of ensuring that the displayed groups possess significant explanatory power with respect to inter-group disparities. The species exhibiting significant disparities in the middle-layer samples of the 50-year-old pit are the most prevalent, among which the species that manifest significant disparities at the phylum level include Patescibacteria and Bacteroidetes. It has been demonstrated that certain strains of Patescibacteria are capable of establishing a symbiotic relationship with *Clostridium* species within the PM environment, with the process of hydrogen transfer within the system promoting the accumulation of acetic acid, butyric acid, and caproic acid [35]. Furthermore, studies have revealed that relevant strains of Bacteroidetes can effectively produce acid and, thereby, enhance the flavor profile of *baijiu* [36]. Concurrently, *Bacteroidetes* have the capacity to degrade carbohydrates and provide organic acid precursors, thus establishing themselves as characteristic microorganisms of high-quality PM [37]. A significant divergence in species composition was observed between *Petrimonas* and *Proteiniphilum* at the genus level in the middle-layer samples of the 50-year-old pit. These two genera are prevalent in old PM and are significantly correlated with various environmental factors in PM [38,39]. LEfSe analysis showed that *Petrimonas* enriched in the middle layer of 50-year-old PM could be used as a signature microorganism for high-quality PM, and combined with the changes in the four organic acids above, its abundance was found to follow the same trend as that of acetic acid and caproic acid content, which provided a new biomarker for the evaluation of the quality of PM.

### 3.4. Differential Analysis of Bacterial Communities in PMs

**Figure 6:**
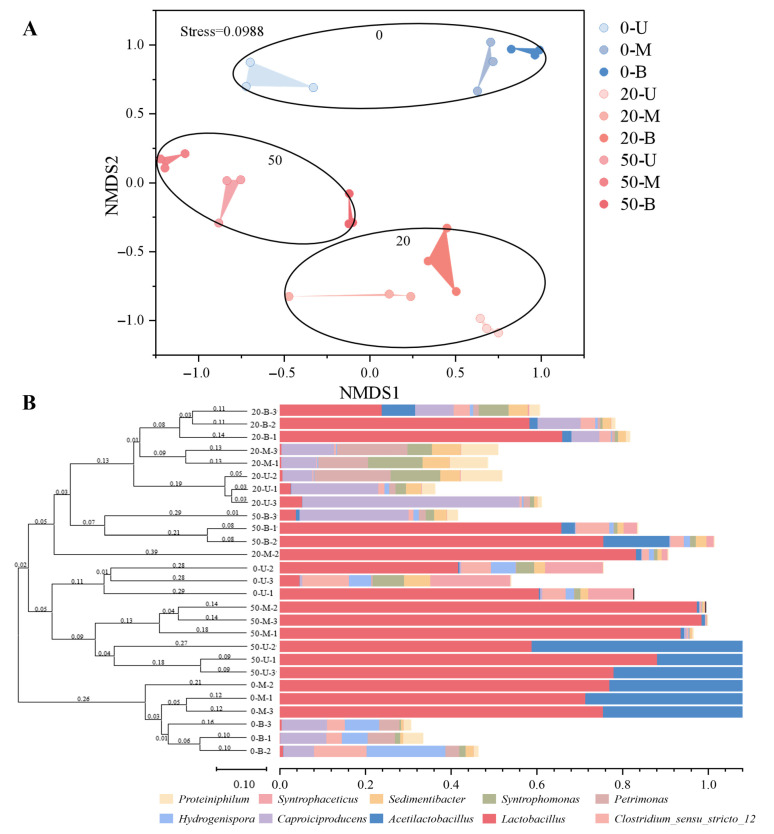
Differential analysis of bacterial communities in PMs. (**A**) Non-metric multidimensional scaling (NMDS) analysis of different PM samples. (**B**) Hierarchical clustering analysis of bacterial communities in different PM samples.

In order to clarify the degree of differences in PM samples of different pit ages and levels, non-metric multidimensional scaling analysis (NMDS) was conducted on the bacterial communities of different samples. The sample distance is indicative of the degree of difference between microbial communities; smaller distances, thus, indicate higher similarity between communities, whilst larger distances indicate significant differences between them. The results of the NMDS analysis, as illustrated in Figure 6A, indicate that the stress value is 0.0988, which is less than 0.2. This finding suggests that the NMDS results are relatively reliable. Furthermore, disparities in PM can be ascertained through hierarchical clustering analysis (Figure 6B), which may be attributable to the evolution of PM microbial communities and alterations in PM maturity. In the early stage of fermentation (0-year-old PM), the microbial community may be in the initial domestication stage, and various microorganisms may not have established a stable ecological balance. Consequently, in comparison with PM in other years, its microbial composition may be more heterogeneous. As the age of the pit increases, the microbial community gradually evolves and matures, resulting in a similar microbial composition in the 20-year-old PM, exhibiting relatively close clustering positions. However, a greater discrepancy is observed between the median and lower quartile of the 50-year-old PM, suggesting that the microbial composition of 50-year-old PM may be undergoing a stable or declining process of change. This alteration may be attributable to the incremental adaptation of certain microbial communities to the prevailing environment, coupled with the expansion of their dominance over an extended period of fermentation. Concurrently, other microorganisms may be in a comparatively weaker state or subject to heightened competitive pressures, resulting in a decline in their population. Consequently, variations in PM samples may be indicative of shifts in microbial community evolution and maturity. As demonstrated by Mao et al. [40], the maturation of PM requires a minimum of 20 years. In the event of inadequate or improper maintenance during the long-term use phase, PM will rapidly enter a degradation period. Furthermore, it is important to note that the physical and chemical properties of the environment also have a significant impact on the clustering results of PM. This is primarily due to the effect that these properties have on the living environment and ecological niche of microorganisms. For instance, in the case of 0-year-old PM, the usage time is comparatively brief, and thus a substantial anaerobic environment has not yet been established. Consequently, it is less influenced by yellow water. This results in a comparable microbial community structure on the walls and bottom of the pit compared to that on the upper part of the pit, given that they are under analogous environmental conditions. It is evident that, in comparison with other PMs, mature PM is capable of establishing a relatively stable anaerobic environment at the base of the pit, a phenomenon attributable to its prolonged fermentation and precipitation. In this environment, the microbial community undergoes a gradual process of adaptation, leading to the establishment of a relatively stable ecological balance. This, in turn, results in the formation of a relatively stable microbial community at the bottom of the pit [41].

### 3.5. Correlation Analysis of Functional Microbial Communities

**Figure 7:**
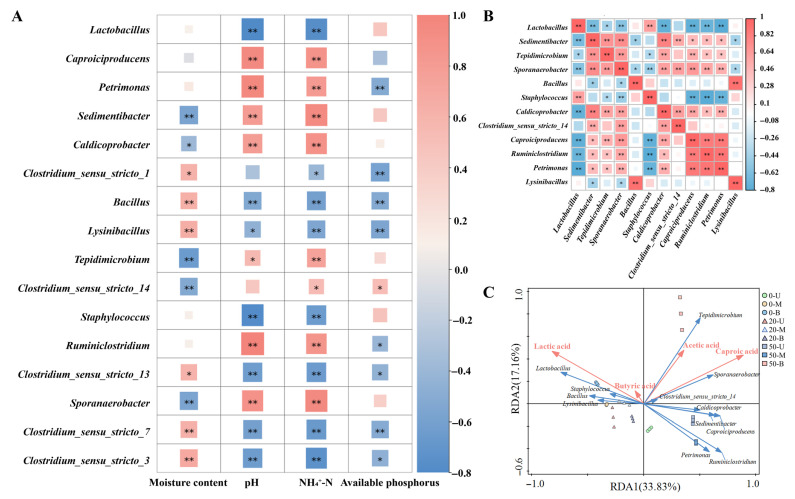
Correlation analysis of functional microbial communities. (**A**) Heat map of the interaction between functional microbial communities and PM physicochemical properties. (**B**) Functional bacterial correlation heat map based on Spearman’s correlation algorithm. (**C**) RDA construction: correlation between functional microbial communities related to caproic acid-producing and lactic acid-utilizing properties and four organic acids. Red in the heat map indicates a positive correlation, and blue indicates a negative correlation (*: *p* < 0.05, **: *p* < 0.01).

Through the literature review, 16 species of “caproic acid-producing and lactic acid-utilizing” bacteria were selected from the microbial community of PM samples, and the heat map of the interaction between functional flora and physical and chemical properties of PM was plotted based on Spearman’s correlation algorithm (Figure 7A). The results demonstrated that PM’s pH and NH_4_^+^-N content exerted the most substantial influences on the functional microbiota. To elaborate further, the following bacterial genera were found to demonstrate a significant positive correlation with PM’s pH: *Caproiciproducens*, *Petrimonas*, *Sedimentibacter*, *Caldicoprobacter*, *Tepidimicrobium*, *Ruminiclostridium,* and *Sporanaerobacter*. The following bacterial genera were found to demonstrate significant negative correlations with PM’s pH: *Lactobacillus*, *Bacillus*, *Lysinibacillus*, *Staphylococcus*, *Clostridium_sensu_stricto_13*, *Clostridium_sensu_stricto_7*, and *Clostridium_sensu_stricto_3*. The following species were found to be significantly positively correlated with NH_4_^+^-N in PM: *Caproiciproducens*, *Petrimonas*, *Sedimentibacter*, *Caldicoprobacter*, *Tepidimicrobium*, *Clostridium_sensu_stricto_14*, *Ruminiclostridium,* and *Sporanaerobacter*. All other species showed negative correlations with NH_4_^+^-N. The interactive correlation heat map provided a visual representation of the interactions between functional communities and environmental factors, thereby analyzing how environmental drivers shape microbial community structure. To illustrate this point, the following example can be considered: The abundance of *Lactobacillus* was found to be significantly and positively correlated with both pH and NH_4_^+^-N, suggesting that elevated pH and NH_4_^+^-N may promote its growth [42]. *Petrimonas* abundance was positively associated with increasing PM pH [31]. *Sedimentibacter* has been shown to have a significant positive correlation with both NH_4_^+^ and available phosphorus [43]. By inferring the relationship between environmental factors and functional flora, the distribution of functional microorganisms in PM can be further verified.

*Baijiu* fermentation is a complex system involving a multi-species co-culture process. The study of microbial interaction is of crucial importance if we are to gain a full understanding of the fermentation process. Furthermore, it is necessary to establish a comprehensive understanding of the relationship between bacterial communities during *baijiu* fermentation. This will provide a valuable reference point for the study of other complex ecosystems [44]. Therefore, in order to elucidate the relationship between bacterial interactions in *nong-xiang baijiu* PM, bacterial interactions were plotted based on the Spearman correlation algorithm by selecting “caproic acid-producing and lactic acid-utilizing” bacteria. The results in Figure 7B show that *Lactobacillus* was significantly negatively correlated with seven species, including *Sedimentibacter*, *Tepidimicrobium*, and *Sporanaerobacter*, and significantly positively correlated with *Staphylococcus*, which was similar to the results of Pang, et al. [45]. The pH change corresponded to the metabolic production of lactic acid in the case of absolute dominance of *Lactobacillus*, which decreased the pH of PM, and the growth of most functional microorganisms was inhibited; when the abundance of *Lactobacillus* was decreased, the pH of PM increased, and the abundance of the community in PM increased. It can be seen that the correlation between *Sedimentibacter*, *Tepidimicrobium*, *Sporanaerobacter*, *Caldicoprobacter,* and the other functional groups varied similarly and all of them had the ability to degrade lactic acid. Similarly, *Caproiciproducens*, *Ruminiclostridium*, and *Petrimonas* all had the ability to produce caproic acid, and their correlation changes are similar to other functional bacterial communities, indicating that some functional bacterial communities with “caproic acid-producing and lactic acid-utilizing” effects have similar abundance trends in different pit mud samples.

Plots of functional bacterial communities in relation to the four organic acids in PM are represented by RDA results. As demonstrated in Figure 7C, a robust correlation exists between microorganisms and organic acids. *Ruminiclostridium*, *Petrimonas*, *Clostridium_sensu_stricto_14*, *Sedimentibacter*, *Caproiciproducens*, *Sporanaerobacter*, and *Caldicoprobacter* were negatively correlated with lactic acid and positively correlated with caproic acid. The metabolic properties of some genera further support this association: for example, *Caproicibacter fermentans* utilizes fructose with final metabolites of acetic, butyric, caproic and lactic acids [46]; *Caproiciproducens galactitolivorans* produces ethanol, acetic, butyric, and caproic acids by anaerobic fermentation, which can directly contribute to caproic acid accumulation [30]; *Sedimentibacter hongkongensis* can ferment amino acids obtained via dipeptide cleavage to ethanol, acetic acid, and succinic acid when co-cultured with other bacteria and its metabolic activities, and it can indirectly promote the growth of caproic acid synthesis-related bacteria by regulating the allocation of environmental carbon sources [47]; and *Petrimonas sulfuriphila* produces acetic acid during glucose fermentation, and acetic acid, as a precursor for caproic acid synthesis, can provide substrate support for caproic acid-producing bacteria [32]. It can, thus, be concluded that the interactions and functional divisions among these microorganisms may regulate and maintain the organic acid content and proportion in PM, thereby affecting the quality and characteristics of PM [48]. Furthermore, the microbial community present within PM exerts a significant influence on the generation and transformation of organic acids. The interplay between microorganisms, characterized by both synergy and competition, exerts a direct influence on the metabolic profile, particularly with regard to the synthesis of caproic acid in *nong-xiang baijiu*. The microorganisms present within the fermentation system may facilitate the production of caproic acid through co-metabolism or mutual promotion of substrate utilization. This process is directly related to the flavor and quality of *baijiu*. Therefore, revealing the relationship between the “caproic acid-producing and lactic acid-utilizing” bacteria and the major organic acids in PM can help to deeply understand the process of PM microbiota changing with the succession of organic acids, and provide a basis for the targeted utilization of microorganisms to strengthen and adjust the microbial community structure of PM.

### 3.6. Prediction of Organic Acid Metabolism Function

**Figure 8:**
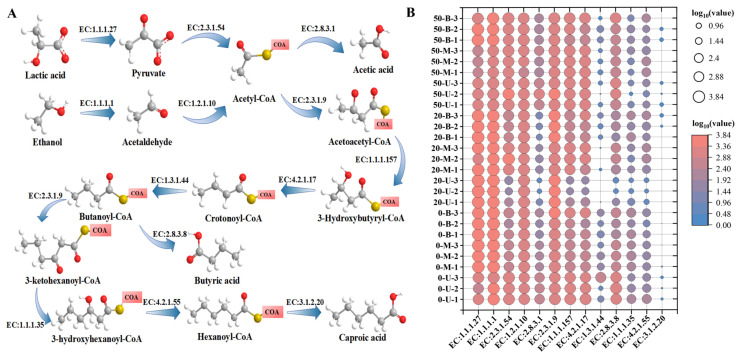
Prediction of organic acid metabolism in PM. (**A**) PICRUSt2 analysis of organic acid metabolism pathways in PM. (**B**) Variations in the abundance of enzymes related to organic acid metabolism in PM samples (The circle size and background color are both mapped to enzyme abundance).

In order to further characterize the metabolic and functional profiles of microbial communities in PM, the PICRUSt2 tool was utilized to predict functional genes encoding key enzymes in organic acid metabolism. As demonstrated in Figure 8A, the metabolic pathways of organic acids in PM are illustrated, accompanied by a statistical analysis of enzymes implicated in lactic, acetic, and butyric acid biosynthesis (Figure 8B). EC:1.1.1.27 (lactate dehydrogenase) catalyzes the redox reaction between pyruvate and lactate. This enzyme was found to be more abundant in 0-year-old PM than in 20-year-old and 50-year-old PM, which is consistent with the higher levels of *Lactobacillus* observed and the increased total acidity found in 0-year-old PM. During the process of anaerobic fermentation, pyruvate is converted into lactate, acetate, or ethanol. Excessive accumulation of lactic acid, a by-product of the fermentation process, has been shown to result in the production of ethyl lactate, which is a compound that can adversely affect the quality of *baijiu*. The reduction in lactic acid accumulation in PM is a critical challenge in the production of *nong-xiang baijiu*. The feasibility of utilizing biofortification to reduce *Lactobacillus* abundance in PM has been demonstrated in previous studies [49]. The abundance of EC:1.1.1.1 (alcohol dehydrogenase) decreased in proportion to increasing pit age in the middle and bottom layers of the pits. This finding indicates a weakened ethanol-to-acetyl-CoA conversion in aged PM. The 50-year-old PM demonstrated higher butyric acid production capacity (EC: 2.8.3.8), which is consistent with the findings of Xia et al. [4]. As illustrated in Figure 4, the abundances of EC:4.2.1.17 and EC:1.1.1.157 increased with pit age in both the middle and bottom layers. It has been established that microorganisms in PM have a propensity to utilize lactic acid as a substrate for the generation of caproic acid, as opposed to other substrates such as ethanol or glucose [50]. Consequently, the enrichment of lactate-degrading bacteria during PM maintenance has been identified as a viable strategy to achieve this objective. Due to the species/strain specificity of fermenting microbial metabolism, the predicted results of this study focus more on revealing the overall metabolic tendencies of the functional flora rather than a precise species-level functional division of labor.

## 4. Conclusions

Through correlation analysis and functional prediction, this study inferred the functional microbial taxa potentially involved in caproic acid-producing and lactic acid-utilizing processes in the PM of *Nong-xiang baijiu*. Among these, *Petrimonas* and *Caproiciproducens* were predicted to be the bacterial taxa involved in caproic acid production, while *Sedimentibacter* was predicted to be associated with lactic acid utilization. These findings facilitate a preliminary understanding of the metabolic characteristics of caproic acid and lactic acid during *Nong-xiang baijiu* fermentation and provide a data reference for subsequent in-depth investigations into the regulatory roles of functional microorganisms. It should be noted that the functional associations identified in this study are based solely on bioinformatic predictions and correlation analyses. The actual metabolic functions of these bacteria require further verification through experiments such as in vitro pure culture, metabolite detection, and metatranscriptomics. Additionally, the related microbial regulatory strategies have not been validated under actual production conditions, and their application value and feasibility in practical brewing need to be clarified through subsequent targeted studies.

## Data Availability

All sequencing data have been deposited at the Sequence Read Archive of the National Center for Biotechnology Information; the accession number is PRJNA1289507. Bio-Sample accessions of bacterial sequences are given in Supplementary Table S3.

## Supplementary Materials

The following supporting information can be downloaded at https://www.mdpi.com/article/10.3390/foods15030416/s1. Figure S1: HPLC spectra of lactic acid, acetic acid, butyric acid, and hexanoic acid standard samples. Figure S2: Standard curves of lactic acid, acetic acid, butyric acid, and hexanoic acid. Table S1: Concentrations of key organic acids in PM samples. Table S2: Statistical table of sequencing quantity of PMs. Table S3: Alpha diversity index table of PMs. Table S4: Table of Bio-Sample accessions of bacterial sequences.
