# Supplementary material for: Revealing the Functional Microbiota of Caproic Acid-Producing and Lactic Acid-Utilizing Bacteria in the Pit Muds for Chinese Nong-Xiang Baijiu Fermentation
Source: Foods. 2026 Jan 23;15(3):416. doi: 10.3390/foods15030416 (PMC12896612; doi:10.3390/foods15030416)
Supplement: Supplementary file 1 [file foods-15-00416-s001.zip › foods-4064414-supplementary.pdf]

Revealing the Functional Microbiota of Caproic Acid-Producing and Lactic Acid-Utilizing Bacteria in the Pit Muds for Chinese *Nong-Xiang Baijiu* Fermentation

Qingwei Feng [1], Xiaohan Li [1], Lijuan Gong [2,3], Yanxia Wei [1], Zhongxue Bai [1], Jian Zhou [1], Yi Ma [2,3,]* and Guiqiang He [1,]*

1 Engineering Research Center of Biomass Materials, Ministry of Education, College of Life Sciences and Agri-Forestry, Southwest University of Science and Technology, Mianyang 621010, China
2 Liquor Making Biotechnology and Application Key Laboratory of Sichuan Province, Sichuan University of Science and Engineering, Yibin 644000, China
3 Liquor Making Biotechnology and Intelligent Manufacturing of Key Laboratory of China National Light Industry, Yibin 644000, China
* Correspondence: zhangyer2008@suse.edu.cn (Y.M.); guiqianghe@swust.edu.cn (G.H.)

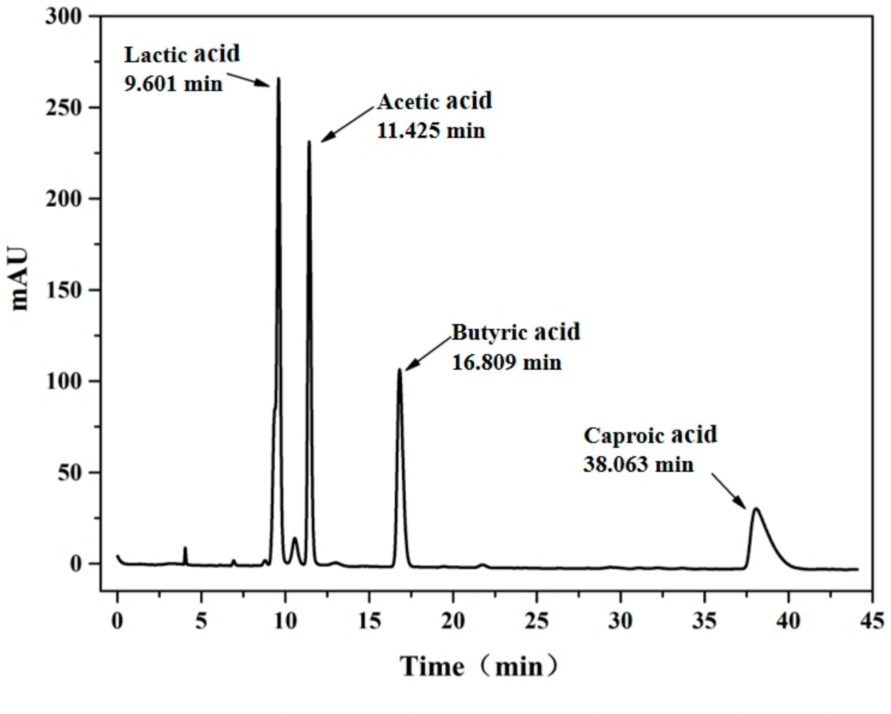

Figure S1. HPLC spectra of lactic acid, acetic acid, butyric acid, and hexanoic acid standard samples.

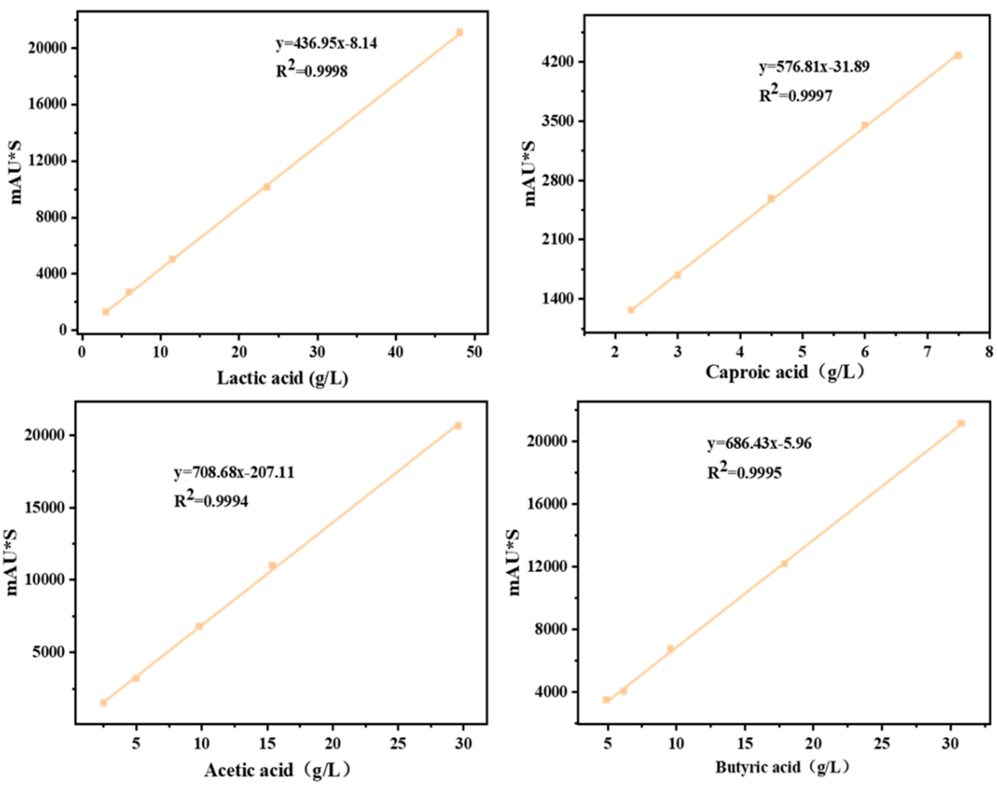

Figure S2. Standard curves of lactic acid, acetic acid, butyric acid, and hexanoic acid.

Table S1. Concentrations of key organic acids in PM samples

Samples	Lactic acid (g/kg)	Acetic acid (g/kg)	Butyric acid (g/kg)	Caproic acid (g/kg)
0-U	23.72 ± 0.60f	7.07 ± 0.06g	1.97 ± 0.26h	3.42 ± 0.14c
0-M	54.74 ± 0.88b	10.36 ± 0.23e	6.78 ± 0.11f	1.53 ± 0.01e
0-B	63.54 ± 0.26a	10.87 ± 0.10d	6.8 ± 0.04f	1.5 ± 0.01e
20-U	37.14 ± 1.35e	7.77 ± 0.05f	8.37 ± 0.15e	2.35 ± 0.30d
20-M	44.77 ± 0.38c	10.52 ± 0.63de	10.18 ± 0.1c	3.25 ± 0.18c
20-B	45.72 ± 0.43c	12.69 ± 0.07b	12.71 ± 0.12a	3.26 ± 0.03c
50-U	20.67 ± 0.26g	10.92 ± 0.01d	3.68 ± 0.08g	6.07 ± 0.12b
50-M	13.14 ± 0.44h	11.77 ± 0.12c	10.56 ± 0.07b	6.53 ± 0.07b
50-B	41.49 ± 0.83d	13.5 ± 0.24a	9.03 ± 0.15d	9.18 ± 0.56a

Note: Samples are labeled by pit age (0, 20, 50 years) and vertical layer (U: Upper layer; M: Middle layer; B: Bottom layer). Data are presented as mean ± standard deviation (n = 3 biological replicates). Different lowercase letters (a–h) in the same column indicate significant differences among samples ($P < 0.05$).

Table S2. Statistical table of sequencing quantity of PMs.

Samples	Input	Filtered	Denoised	Merged	Non-chimeric	Non-singleton
	143074	129970	126994	114118	88268	87651
0-U	136882	125294	122339	112418	89506	89179
	137292	126208	123328	113972	87799	87312
	105561	95076	93976	92767	91239	91209
0-M	96471	86811	86439	85832	83204	83176
	127444	114905	114348	113043	110213	110173
	105092	95145	94599	94217	93524	93503
0-B	129941	117212	116693	115684	113649	113581
	124150	112532	111667	109707	104753	104664
	136293	123562	122551	121298	119007	118868
20-U	146271	132531	131997	130981	130054	130018
	147003	133074	132542	131674	129857	129810
	142476	130329	126392	113108	79501	78781
20-M	143256	132962	129395	117591	88620	8805
	136300	124851	120859	106855	76178	75631
	133601	122100	121314	120060	118173	118080
20-B	138747	127735	126909	12562	121007	120939
	134886	124218	123285	122179	119512	119437
	133691	123968	121609	113545	100869	100629
50-U	138587	128082	125831	119661	91137	90922
	136290	126554	123106	111106	95871	95579
	142476	130329	126392	113108	79501	78781
50-M	143256	132962	129395	117591	88620	8805
	136300	124851	120859	106855	76178	75631
	135089	125635	124240	121647	115128	115006
50-B	143575	133461	132404	130208	125848	125764
	143983	132796	132217	130850	127975	127941

Table S3. Alpha diversity index table of PMs.

Samples	Shannon	Simpson	Chao1	Observed_species	Goods_coverage	Faith_pd
	7.33415	0.981424	1266.58	1227.4	0.99811	73.9452
0-U	6.23789	0.937514	1093.63	1058.6	0.998378	80.6446
	5.49665	0.894447	1079.14	1048.8	0.998413	76.0416
	4.62706	0.790107	477.796	469.1	0.999694	29.3825
0-M	5.65724	0.87976	426.652	415.2	0.999804	22.0992
	4.13149	0.756955	476.509	455.3	0.999552	32.5563
	3.54594	0.651112	339.671	333.6	0.999808	22.8247
0-B	2.64814	0.555386	438.856	422.7	0.999479	30.3996
	4.73827	0.804035	556.909	538	0.999464	27.6582
	1.65371	0.331742	624.53	573	0.998604	42.3182
20-U	0.928622	0.206301	254.782	229.7	0.99941	22.9882
	1.25915	0.322215	285.402	266.5	0.999378	23.9348
	3.6343	0.693406	419.437	396.3	0.999266	35.0653
20-M	5.39871	0.957092	323.998	311	0.999648	24.5871
	4.54675	0.858754	323.627	312.8	0.999669	26.9225
	2.98558	0.633538	630.414	602.7	0.999122	47.1976
20-B	3.95934	0.803149	569.199	539.5	0.999125	42.9061
	4.27544	0.80719	688.715	661.2	0.999222	60.2699
	6.64895	0.967138	936.351	885.5	0.998539	64.6558
50-U	3.9856	0.774461	784.939	746.2	0.998539	53.1167
	6.90981	0.974456	1039.35	984.2	0.998214	72.6082
	6.6919	0.963282	1206.08	1178.8	0.9982	75.0483
50-M	6.51908	0.964573	1126.09	1080.4	0.998028	67.917
	6.72298	0.964483	1139.05	1119.2	0.998569	67.8419
	3.87791	0.70251	744.449	709.2	0.998861	51.1441
50-B	4.22255	0.765533	740.136	690.8	0.998806	50.9122
	5.45226	0.946402	445.022	420.9	0.999523	36.4983

Table S4. Table of Bio-Sample accessions of bacterial sequences.

Sample names	Bio-Sample accessions	Sample names	Bio-Sample accessions
0-U-1	SAMN49893456	20-B-1	SAMN49893471
0-U-2	SAMN49893457	20-B-2	SAMN49893472
0-U-3	SAMN49893458	20-B-3	SAMN49893473
0-M-1	SAMN49893459	50-U-1	SAMN49893474
0-M-2	SAMN49893460	50-U-2	SAMN49893475
0-M-3	SAMN49893461	50-U-3	SAMN49893476
0-B-1	SAMN49893462	50-M-1	SAMN49893477
0-B-2	SAMN49893463	50-M-2	SAMN49893478
0-B-3	SAMN49893464	50-M-3	SAMN49893479
20-U-1	SAMN49893465	50-B-1	SAMN49893480
20-U-2	SAMN49893466	50-B-2	SAMN49893481
20-U-3	SAMN49893467	50-B-3	SAMN49893482
20-M-1	SAMN49893468		
20-M-2	SAMN49893469		
20-M-3	SAMN49893470		
